# Analysis of vaccination campaign effectiveness and population immunity to support and sustain polio elimination in Nigeria

**DOI:** 10.1186/s12916-016-0600-z

**Published:** 2016-03-30

**Authors:** Alexander M. Upfill-Brown, Arend Voorman, Guillaume Chabot-Couture, Faisal Shuaib, Hil M. Lyons

**Affiliations:** Institute for Disease Modeling, Bellevue, WA USA; Bill & Melinda Gates Foundation, Seattle, WA USA; Federal Ministry of Health, Federal Republic of Nigeria, Abuja, FCT Nigeria; National Polio Emergency Operations Center, Abuja, FCT Nigeria

**Keywords:** Campaigns, Coverage, Eradication, Hierarchical modeling, Immunity, Nigeria, Polio, Supplementary immunization activities

## Abstract

**Background:**

The world is closer than ever to a polio-free Africa. In this end-stage, it is important to ensure high levels of population immunity to prevent polio outbreaks. Here, we introduce a new method of assessing vaccination campaign effectiveness and estimating immunity at the district-level. We demonstrate how this approach can be used to plan the vaccination campaigns prospectively to better manage population immunity in Northern Nigeria.

**Methods:**

Using Nigerian acute flaccid paralysis surveillance data from 2004–2014, we developed a Bayesian hierarchical model of campaign effectiveness and compared it to lot-quality assurance sampling data. We then used reconstructed sero-specific population immunity based on campaign history and compared district estimates of immunity to the occurrence of confirmed poliovirus cases.

**Results:**

Estimated campaign effectiveness has improved across northern Nigeria since 2004, with Kano state experiencing an increase of 40 % (95 % CI, 26–54 %) in effectiveness from 2013 to 2014. Immunity to type 1 poliovirus has increased steadily. On the other hand, type 2 immunity was low and variable until the recent use of trivalent oral polio vaccine. We find that immunity estimates are related to the occurrence of both wild and vaccine-derived poliovirus cases and that campaign effectiveness correlates with direct measurements using lot-quality assurance sampling. Future campaign schedules highlight the trade-offs involved with using different vaccine types.

**Conclusions:**

The model in this study provides a novel method for assessing vaccination campaign performance and epidemiologically-relevant estimates of population immunity. Small-area estimates of campaign effectiveness can then be used to evaluate prospective campaign plans. This modeling approach could be applied to other countries as well as other vaccine preventable diseases.

**Electronic supplementary material:**

The online version of this article (doi:10.1186/s12916-016-0600-z) contains supplementary material, which is available to authorized users.

## Background

The Global Polio Eradication Initiative (GPEI) is closer than ever to a polio-free world. This success can be attributed in part to the use of supplementary immunization activities (SIAs) or campaigns, at the largest scale ever seen in public health [1, 2]. In order to achieve poliovirus (PV) eradication, it is crucial for policymakers to know which areas are most vulnerable to PV and what impact SIAs are having in addressing this vulnerability.

During a SIA, health workers attempt to provide oral polio vaccine (OPV) to all children under 5 years old in a given area, typically through house-to-house vaccination campaigns. Reaching all children is difficult for a variety of reasons, including out-of-house children, vaccine refusals, and incomplete household maps. Currently, two types of post-campaign surveys are used to assess campaign coverage: independent monitoring [3], and cluster lot quality assurance sampling (LQAS) [4]. However, coverage estimates from independent monitoring are often unrealistically high and inconsistent with observed epidemiology [5]. On the other hand, coverage estimates from LQAS are considered more accurate, but are less precise and not as widely available. Further, both LQAS and independent monitoring are household-based surveys, and may miss children in informal settlements, hard-to-reach areas, and mobile populations [4].

In order to gauge the need for SIAs, policymakers use indicators of vulnerability based primarily on dose-histories of non-polio Acute Flaccid Paralysis (NP-AFP) cases collected through the global polio surveillance network [6]. While reported dose histories may suffer from recall bias, they have been shown to be robust indicators of individual immunity [7, 8], and the collection of dose-histories from AFP cases in a particular area serve as useful indicators of population immunity [9]. However, sparse data limits accuracy of vulnerability estimates at small spatial scales [9], and also the responsiveness of the estimates to immunization activities. Perhaps more importantly, reported doses are the result of past SIAs, but the impact of SIAs on reported doses is not directly measured or used in SIA planning.

In this manuscript, we introduce a novel method for estimating campaign quality, which we call campaign effectiveness, from dose histories of non-polio AFP data in northern Nigeria, and demonstrate how these quality estimates can be used to estimate population immunity. We use the Bayesian hierarchical model to account for sparse data and improve estimates at the district level. We then show how these quality estimates and immunity can be used prospectively in SIA planning. We validate our method by comparing estimated population immunity to the occurrence of confirmed wild poliovirus (WPV) and circulating vaccine-derived poliovirus (cVDPV) cases, and by comparing campaign effectiveness estimates to LQAS data.

## Methods

### Data

#### Acute Flaccid Paralysis (AFP) database

The global polio surveillance network detects AFP cases of any cause [10]. Surveillance officers collect stool samples from each case, which are tested in order to determine whether paralysis was caused by PV. Surveillance officers also collect basic demographic information on each AFP case, including age, sex, date of onset, and number of polio vaccinations received. Importantly, this information is collected before the cause of paralysis is known. The vast majority of AFP cases are classified as NP-AFP and serve as the basis of our analysis. In this analysis, we used the Nigerian AFP database and the LQAS database maintained by the Nigerian country office of the World Health Organization (WHO).

#### Lot quality assurance sampling (LQAS)

Following a vaccination campaign in which vaccine recipients had their fingers marked by vaccinators, independent surveyors visited six randomly chosen villages within a district and checked for the presence of finger-marking on 10 children [4]. Only a subset of districts participating in a vaccination campaign were visited by LQAS surveyors. In the course of a year, most districts were visited by surveyors at least one time. We included LQAS surveys from 2009 through 2015 in our analysis.

#### Supplementary immunization activities (SIA) database

The dose-histories of NP-AFP cases are referenced against the SIA database maintained by the Nigerian WHO. This records basic information for each polio vaccination campaign, including the date and location of the campaign, which vaccines were used, and which age groups were targeted. Case date of onset, age, and district were used herein to determine campaigns that could have contributed to reported doses.

#### Vaccine efficacy

There are five different formulations of OPV: trivalent OPV (tOPV), bivalent OPV (bOPV), and monovalent OPV (mOPV) for each serotype of PV (1, 2, or 3). tOPV contains antigen for types 1, 2, and 3 PV, while bOPV contains antigen for only types 1 and 3 PV. Each vaccine has a different associated efficacy against each serotype, which may vary with socio-economic context [7, 8, 11]. In our analysis, we used vaccine efficacy estimated by comparing dose-histories of polio and NP-AFP cases in northern Nigeria [8].

Institutional ethics approval was not sought for AFP surveillance and LQAS monitoring data as they are retrospective and anonymized.

### Overview of statistical analysis

**Fig. 1:**
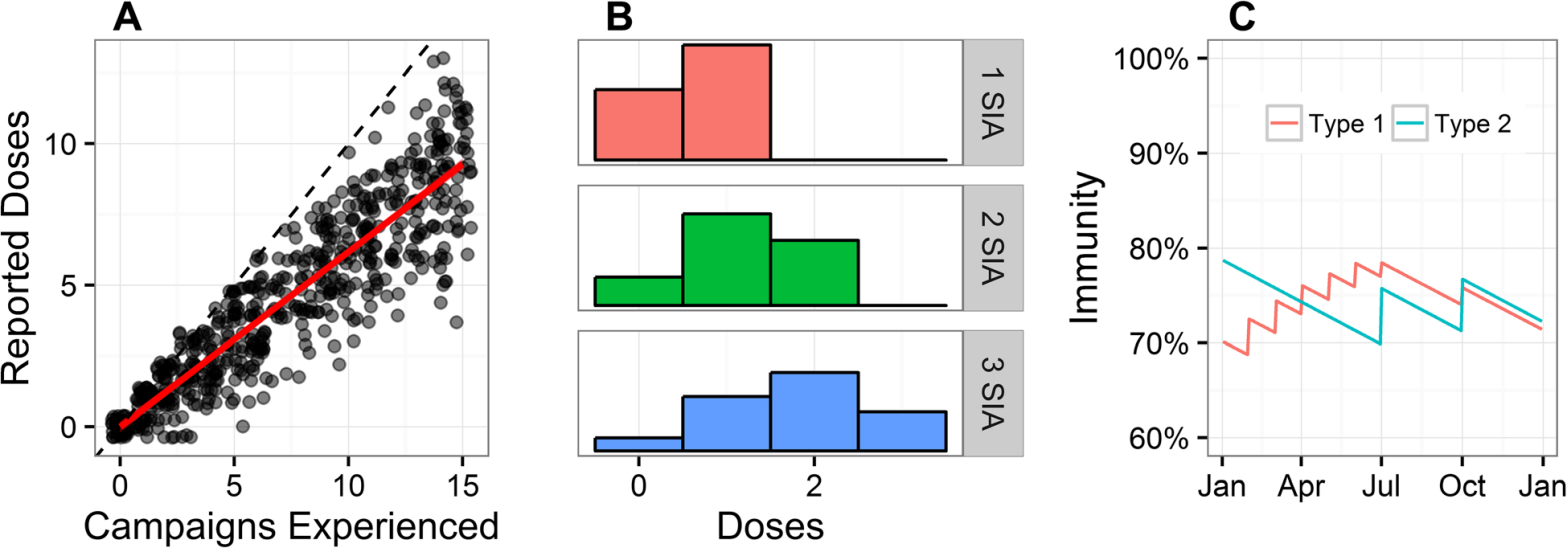
Overview of the modeling procedure. (**a**) We first estimated campaign effectiveness, a measure of how reported doses respond to campaigns. (**b**) Using campaign effectiveness, we estimated the number of doses reported by a child, given the number of SIAs experienced. (**c**) We estimated immunity based on the 6- to 59-month-old age distribution, the number of doses for a given age, and vaccine effectiveness

Figure 1 provides a visual overview of the statistical procedure. The first step in the process was estimating campaign effectiveness through NP-AFP data by comparing the reported doses – observed with error – with campaigns experienced (Fig. 1a). This effectiveness and an assumption of random, independent participation induces a distribution of doses for the population of interest as well as subgroups of interest. Generally, the more SIAs experienced, the more doses received (Fig. 1b); in particular, a SIA changes the dose distribution in the population over the short period in which it was executed. This change in the dose distribution is accompanied by a change in the immune fraction by serotype, related to the efficacy of the vaccine used and the campaign effectiveness (Fig. 1c).

#### Bayesian hierarchical modeling of campaign effectiveness

We considered model campaign effectiveness at the district level, called local government areas (LGA) in Nigeria. LGAs are an administrative level lower than province or states in Nigeria and are particularly meaningful units of analysis as many polio eradication operations (including vaccination and monitoring) are organized by LGAs.

We specified a Bayesian hierarchical model of campaign effectiveness to account for temporal patterns and between-LGA differences, with the aim of producing smoothed estimates by LGA and year. Let *p*_*ijta*_ be the campaign effectiveness for state *i*, LGA *j*, year *t*, and age stratum *a*. We modeled yearly LGA-level campaign effectiveness by age *p*_*ijta*_ with

$$ \log \mathrm{it}\left({p}_{ijta}\right)={\beta}_{ia}+{b}_{ij}+{u}_{it}+{v}_{ijt}, $$

where *β*_*ia*_ is an age effect, *b*_*ij*_ ~ *N*(0, *σ*_*i*_^2^) is a random effect for LGA, and [*u*_*i*1_, …, *u*_*iT*_]^*T*^ ~ *N*_*T*_(0, *Σ*(*σ*_*iu*_^2^)) and [*v*_*ij*1_, …, *v*_*ijT*_]^*T*^ ~ *N*_*T*_(0, *Σ*(*σ*_*iv*_^2^)) are first order normal random walk priors for state and LGA temporal variation, respectively [12]. The index *i* appears in the subscript for parameters as we executed separate models for each state *i*. Priors for age effects and hyperpriors for variance parameters governing the random effects are diffuse; details of Bayesian specification may be found in Additional file 1.

We used a negative binomial distribution to model reported doses per child, where the expected value (mean) is the sum of campaign effectiveness across campaigns experienced by the child. The negative binomial distribution allows the variance to be flexibly fit in the estimation procedure, which may accommodate imprecise recall and heterogeneous vaccination coverage (further discussion of model details can be found in Additional file 1).

We assessed three models of different complexity. The full model was as specified above; other models considered were nested within this full model, removing age and then the district random walks. The Deviance Information Criterion was used to pick the model that best balanced fit and complexity [13].

#### Campaign-derived immunity

Polio has three serotypes, and high population immunity to each type is key to achieving elimination. Vaccines have different efficacies for different types [8]. Variable campaign effectiveness, the SIA database, and vaccine efficacies can be used to model the time course of expected immunity for an individual and the population of interest.

Let *ϕ*_*k*_ be the efficacy – the probability of seroconversion – of the vaccine used in the *k*th campaign experienced by a child of age *a* by time *t*, and *p*_*k*_ be the associated campaign effectiveness. With independent participation in campaigns and independent seroconversion, the probability of vaccine-based seroconversion for the child given all the campaigns experienced is

$$ I\left(t,a\right)=1-{\displaystyle \prod_k\left(1-{p}_k{\phi}_k\right)}. $$

Population immunity can then be calculated by integrating over an age distribution *F*: I(t) = ∫*I*(*t*, *a*)*dF*(*a*). Computational methods may be found in Additional file 1.

#### Implementation

All analyses were performed in R [14]. We used MC-STAN [15, 16] to obtain samples from the posterior distribution of campaign effectiveness *p*_*ijta*_ for each campaign in each LGA, following the model described above. Using these posterior samples, we estimated effectiveness through the posterior mean and summarized uncertainty with 95 % credible intervals. We approximated the posterior distribution of functions of LGA-level campaign effectiveness, such as state-wide campaign effectiveness and immunity, by applying these functions to the posterior samples. As with campaign effectiveness, we summarized these distributions through their posterior means and 95 % credible intervals.

## Results

### Campaign effectiveness

**Fig. 2:**
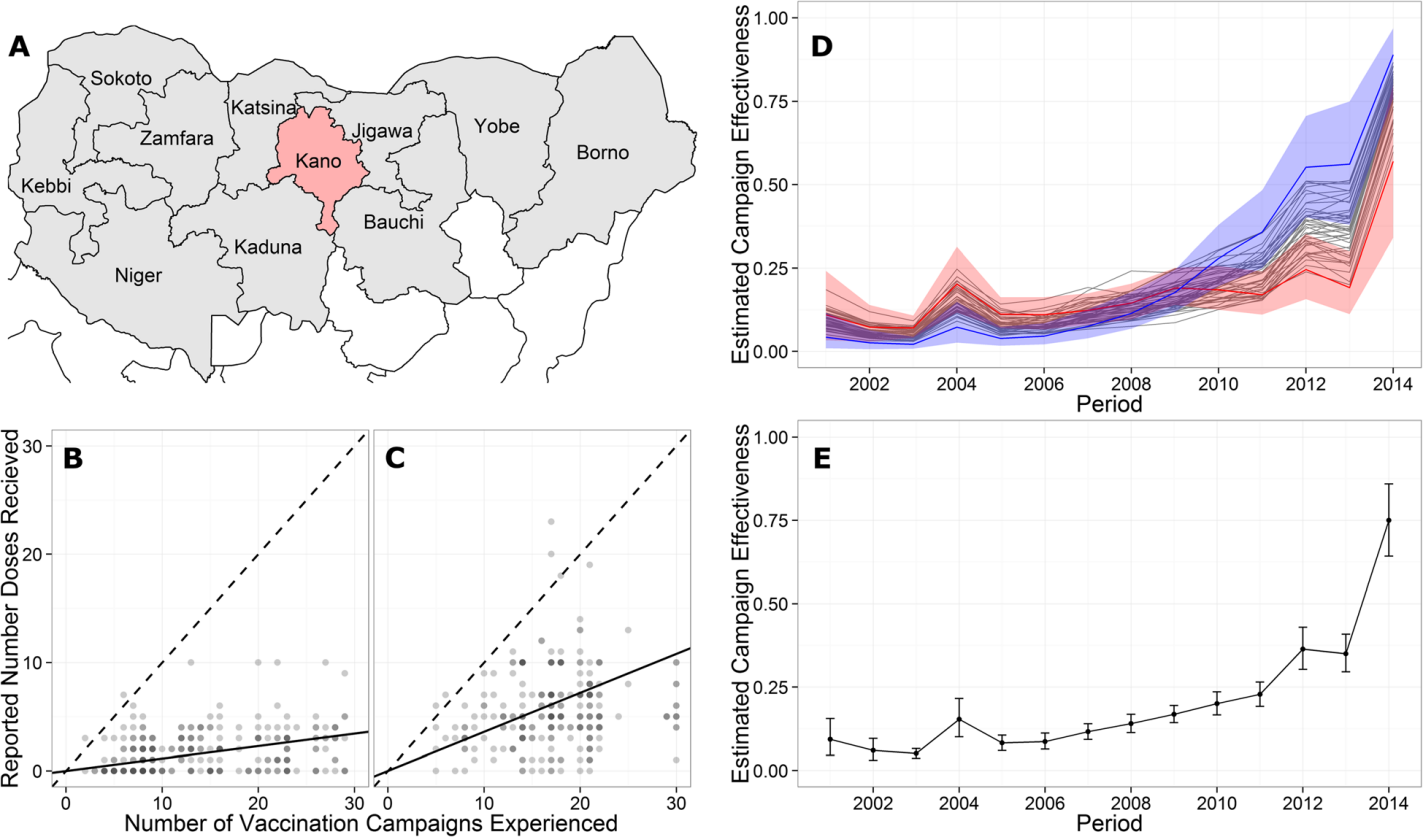
Kano campaign effectiveness. (**a**) Map showing location of Kano state with 10 other high-risk states in northern Nigeria. (**b**, **c**) Reported number of doses received relative to estimated number of campaigns experienced for NP-AFP cases in Kano born in 2004 (**b**) and 2010 (**c**). (**d**) District traces of modeled campaign effectiveness with highest and lowest performing districts in 2014 colored red and blue, respectively. Shaded regions represent 95 % credible intervals (CI). (**e**) Kano state average campaign effectiveness by time period with 95 % CI

Studying data from Kano state (Fig. 2a) first, we found that the relationship between reported number of doses received and the number of campaigns experienced has strengthened over time: for children born in 2004, the rate was 0.11 doses per campaign experienced (Fig. 2b), while for children born in 2010, this rate was 0.36 (Fig. 2c). Model results indicate that campaign effectiveness shows a steady increase, before it increases rapidly in 2014 (Fig. 2d). The maximum district campaign effectiveness achieved in 2014 was 89 % (95 % CI, 76–97 %), while the minimum was 56 % (34–80 %). Four years prior, the best performing district was achieving 30 % (20–42 %) effectiveness, while the worst was achieving 13 % (7–19 %). State average campaign effectiveness (calculated based on population-weighted district estimates) increased 40 % between 2013 and 2014, from 35 % (30–41 %) to 75 % (64–86 %) (Fig. 2e).

**Fig. 3:**
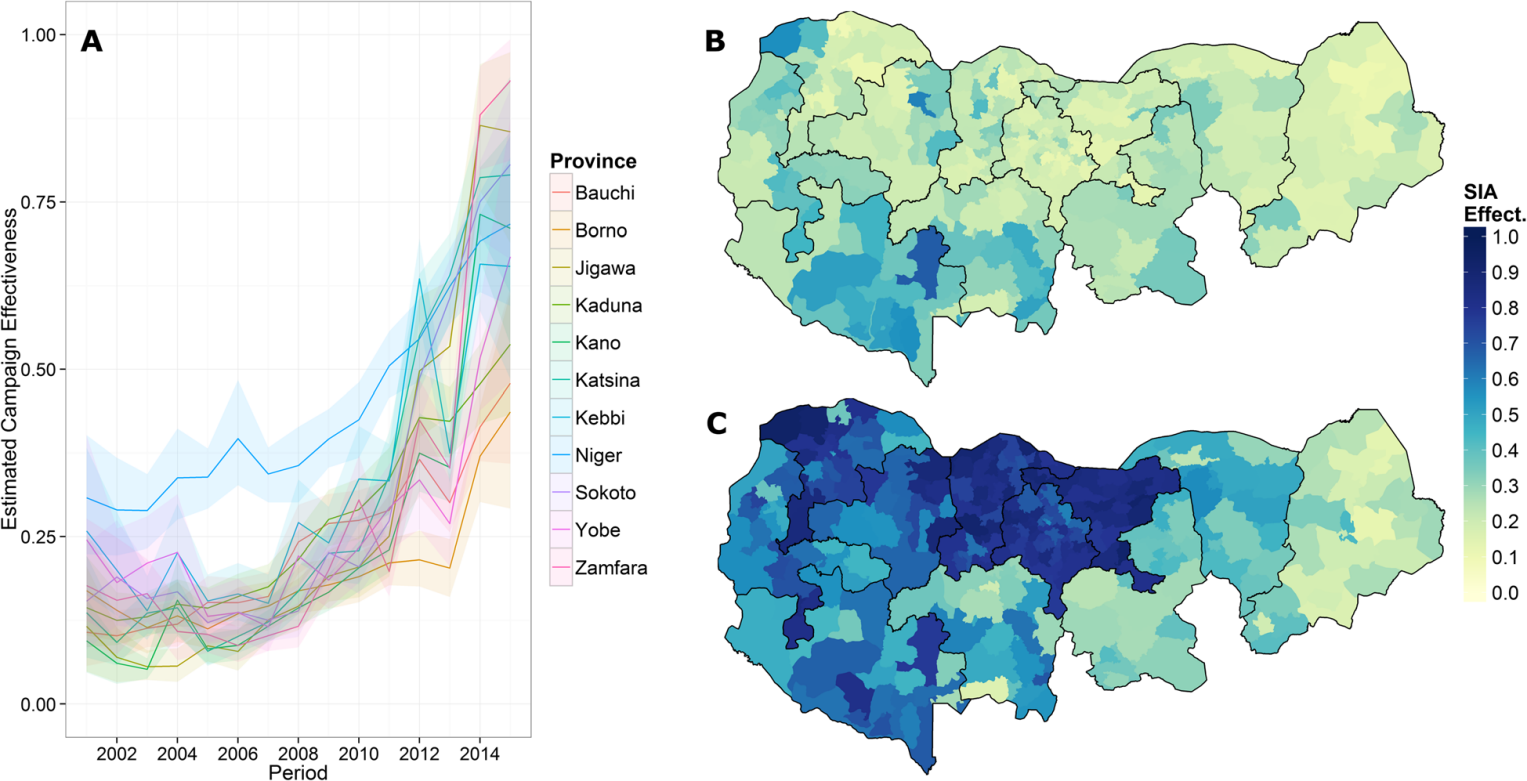
Northern state campaign effectiveness. (**a**) Mean campaign effectiveness estimates for northern high-risk states. Shaded region represents pointwise 95 % credible interval. (**b**, **c**) Estimated district campaign effectiveness in 2010 (**b**) and 2014 (**c**)

Campaign effectiveness increased across states in northern Nigeria since 2004 (Fig. 3a–c). Despite improvements, campaign effectiveness remained low in Borno, Bauchi, and parts of Yobe and Kaduna. On average, campaign effectiveness remained below 50 % in these four states in 2014. Campaign effectiveness increased by more than 40 % between 2010 and 2014 in Jigawa, Kano, Katsina, Sokoto, and Zamfara (Additional file 1).

The age-based participation effects in the model were similar across states, and suggested older children were less likely to report doses from additional campaigns. In Kano, relative to 6- to 11-month-olds, 1-year-olds (12–23 months) had 1.2 (0.9–1.6) times lower odds of reporting a dose from a campaign, 2-year-olds had 6.2 (3.6–13.5) time lower odds, 3-year-olds had 2.3 (1.6–3.4) times lower odds, while 4-year-olds had 2.8 (1.5–7.0) lower odds (Additional file 1).

**Fig. 4:**
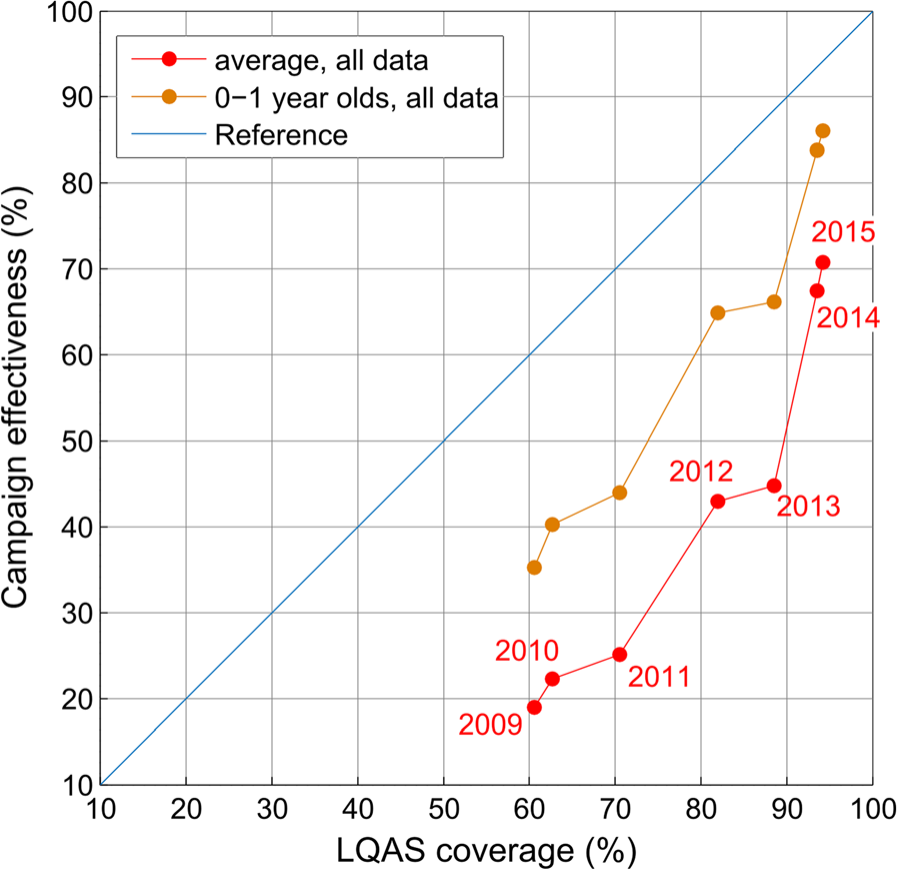
Comparison of calculated campaign effectiveness and LQAS data, using 5,770 LQAS lots matched to calculated campaign effectiveness. Curves show average campaign effectiveness and average LQAS coverage, by year. The red curve shows the mean campaign effectiveness across all age groups compared to the LQAS coverage, whereas the orange curve shows the campaign effectiveness for the 0- to 1-year-old age group specifically. The distance of the data from the blue reference curve shows the bias of the calculated campaign effectiveness compared to LQAS data

By comparing 5,770 LQAS lots collected across northern Nigeria, from 2009–2015, with matched campaign effectiveness, we found a robust relationship between the two measures of SIA coverage. The global correlation coefficient between LQAS coverage and campaign effectiveness was 0.42, capturing 18 % of the variance in the LQAS data; both measures showed increasing coverage across the north of Nigeria and campaign effectiveness was on average 33 % below the corresponding LQAS value (i.e. it was biased with respect to the LQAS data). The difference between average LQAS coverage and average campaign effectiveness decreased to 15 % and their correlation increased to 0.45 if we considered campaign effectiveness for the 0- to 1-year-old age group only (Fig. 4 and Additional file 1).

### Reconstructing immunity

**Fig. 5:**
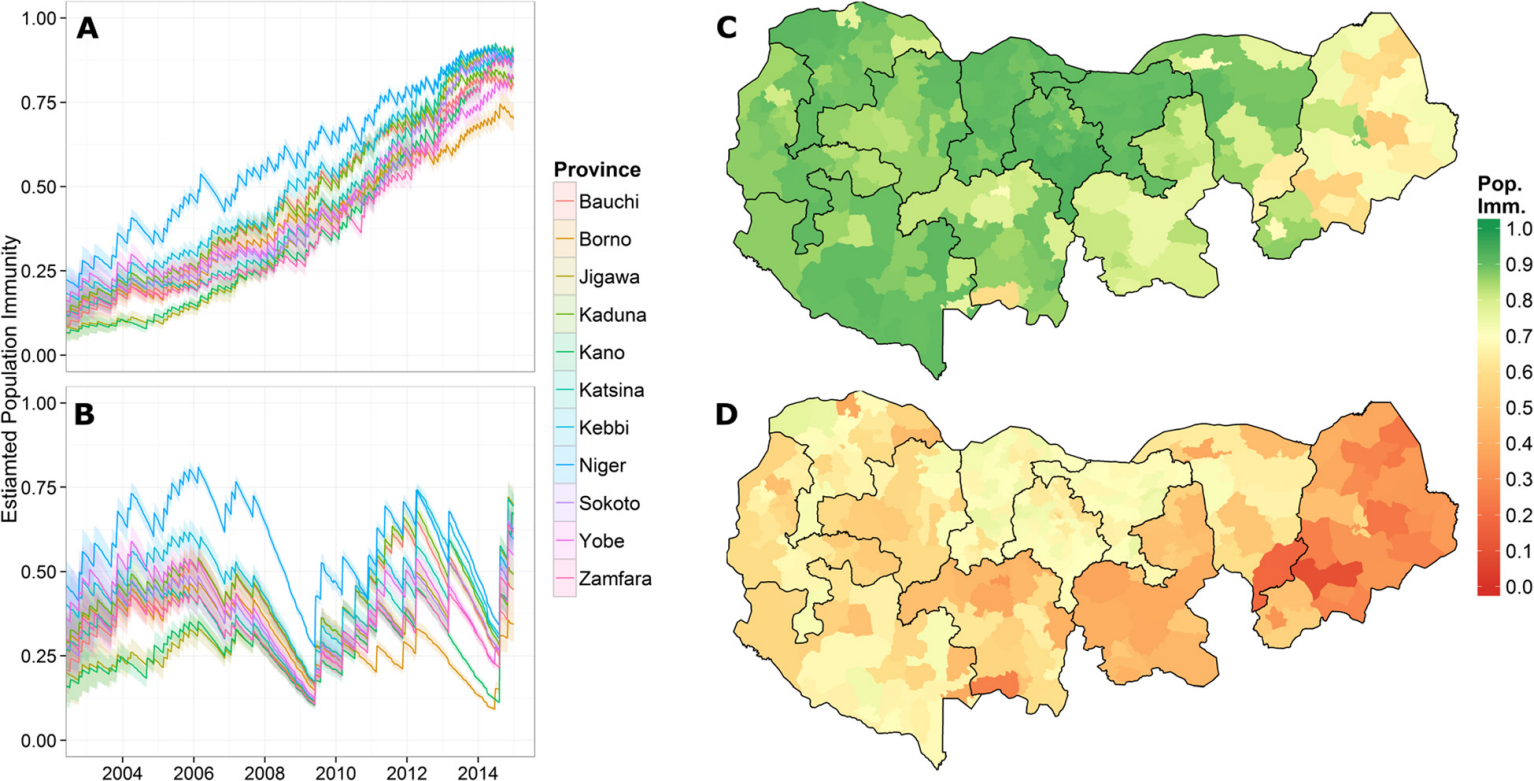
Northern state reconstructed population immunity. (**a**, **b**) Average type 1 (**a**) and type 2 (**b**) population immunity for northern states, shaded region represents pointwise 95 % credible interval. (**c**, **d**) Estimate district-level type 1 (**c**) and type 2 (**d**) population immunity as of December 31st, 2014

Using model outputs of campaign effectiveness, along with the historical campaign calendar and estimated vaccine effectiveness, we reconstructed sero-specific population immunity (Fig. 5). Type 1 immunity increased consistently over time across all states (Fig. 5a), while type 2 immunity decreased substantially after 2012 until the latter half of 2014, when tOPV (containing type 2 vaccine) was used for the first time in more than a year (Fig. 5b). The saw-tooth pattern captures the spike in population immunity immediately following a campaign, and the decay in immunity appeared as older children left the target age group and unimmunized infants replaced them.

District immunity estimates were significantly associated with the subsequent presence or absence of WPV1 and cVDPV2. We related the estimated immunity at the beginning of a 6-month period to the presence or absence of a case(s) of PV in 2004 through 2014. Average type 1 immunity was 29 % in districts that reported at least one WPV case in the following 6 months compared to 53 % in districts that did not (*P* <0.001, using a two-sided *t*-test). Furthermore, we found that 0.9 % of districts with estimated type 1 immunity above 80 % reported a WPV1 case in the following 6 months. Average type 2 immunity was 27 % in districts that reported at least one cVDPV2 case in the following 6 months compared to 39 % in districts that did not (*P* <0.001). Additionally, only 0.6 % of districts with estimated type 2 immunity above 50 % reported a cVDPV2 case in the following 6 months (Additional file 1).

District-level immunity estimates (Fig. 5c,d) suggested that, as of the end of 2014, all districts in Jigawa, Kano, Katsina, Kebbi, and Zamfara had greater than 80 % average type 1 population immunity. In Niger and Sokoto, only one district was below 80 %, while Bauchi and Kaduna had six districts each below 80 % immunity. In Borno and Yobe, states with inaccessible districts, there were 21 and six districts, respectively, below 80 %.

Our approach allowed full propagation of uncertainty in campaign effectiveness into immunity calculations (see shaded regions, Fig. 5a,b). Prediction intervals could then be used to assess which districts were significantly below or above a target immunity threshold. For example, we found that only Borno (11 districts), Yobe (3), and Kaduna (2) had estimated type 1 population immunity significantly below 80 % as of the end of 2014 (i.e. posterior 95 % CI is below the target immunity level).

### Evaluation of proposed campaign calendars

Campaign effectiveness estimates can be used to project future immunity given a set of planned campaigns. As an example, we compared three different future campaign calendars for northern Nigeria in advance of the removal of tOPV from vaccination globally in April 2016 [17]. All districts experienced vaccination rounds of bOPV, tOPV, tOPV, and bOPV in January, March, April, and June, respectively. Beginning in the second half of 2015, there were three potential calendars (Table 1). One calendar represents the planned schedule (‘Planned’), one includes more bOPV in place of tOPV (‘bOPV’), and the last includes only tOPV campaigns (‘tOPV’).

**Table 1:** Future potential campaign calendars

Name	July-15	Sept-15	Oct-15	Dec-15	Jan-16	Feb-16	Mar -16	Apr-16	Jun-16
Planned	tOPV	tOPV	tOPV	bOPV	bOPV	tOPV	tOPV	bOPV	bOPV
bOPV	tOPV	bOPV	tOPV	bOPV	bOPV	tOPV	bOPV	bOPV	bOPV
tOPV	tOPV	tOPV	tOPV	tOPV	tOPV	tOPV	tOPV	bOPV	bOPV

Each of the three hypothetical campaign calendars uses a different combination of tOPV and bOPV campaigns

b, bivalent; OPV, Oral polio vaccine; t, trivalent

**Fig. 6:**
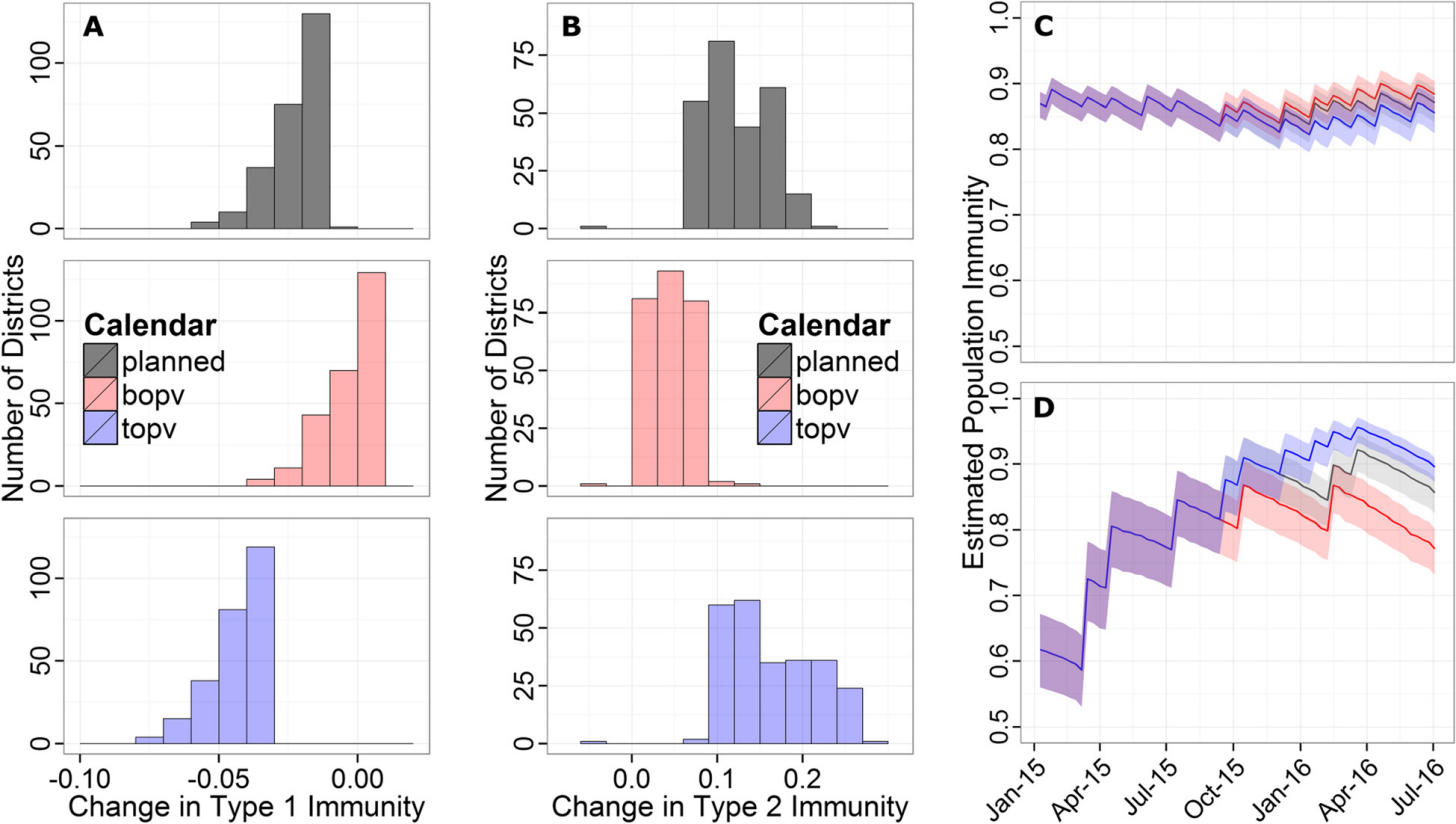
Impact of different future campaign schedules. (**a**, **b**) Distributions of the change in type 1 (**a**) and type 2 (**b**) district immunity under three different campaign calendars (Table 2) from June 20th, 2015, to April 10th, 2016 (immediately following the global cessation of the use of trivalent oral polio vaccine). (**c**, **d**) Projected type 1 (**c**) and type 2 (**d**) population immunity for Zamfara state under the three different campaign calendars

The impact of these three calendars is compared in Fig. 6 and Table 2. In the planned calendar, almost all districts would experience a small drop in type 1 immunity with a corresponding 10 % increase in type 2 immunity (Fig. 6a,b). Under the bOPV calendar, the average district would maintain the same level of type 1 immunity, but the corresponding increase in type 2 immunity would be reduced to 5 %. Finally, under the tOPV calendar, there would be a 4 % drop in type 1 immunity in most districts, but the average increase in type 2 immunity would be 15–20 %.

**Table 2:** Northern state projected type 2 immunity

Province	Before^a^	Planned^b^	bOPV^b^	tOPV^b^
Bauchi	0.57 (0.50–0.63)	0.74 (0.68–0.80)	0.64 (0.58–0.70)	0.81 (0.75–0.86)
Borno	0.37 (0.31–0.43)	0.38 (0.33–0.44)	0.34 (0.29–0.40)	0.41 (0.36–0.46)
Jigawa	0.84 (0.79–0.87)	0.93 (0.91–0.95)	0.87 (0.83–0.89)	0.96 (0.95–0.97)
Kaduna	0.60 (0.54–0.67)	0.77 (0.69–0.83)	0.67 (0.60–0.74)	0.82 (0.76–0.88)
Kano	0.83 (0.77–0.87)	0.92 (0.89–0.94)	0.85 (0.82–0.88)	0.95 (0.93–0.96)
Katsina	0.83 (0.79–0.86)	0.93 (0.91–0.94)	0.86 (0.83–0.88)	0.96 (0.94–0.96)
Kebbi	0.73 (0.68–0.78)	0.88 (0.84–0.91)	0.80 (0.75–0.83)	0.92 (0.89–0.95)
Niger	0.74 (0.68–0.79)	0.87 (0.82–0.91)	0.79 (0.73–0.83)	0.91 (0.86–0.94)
Sokoto	0.77 (0.73–0.81)	0.90 (0.87–0.92)	0.82 (0.79–0.85)	0.94 (0.92–0.95)
Yobe	0.62 (0.55–0.70)	0.73 (0.67–0.79)	0.65 (0.59–0.71)	0.78 (0.73–0.83)
Zamfara	0.78 (0.72–0.83)	0.91 (0.88–0.93)	0.83 (0.79–0.86)	0.94 (0.92–0.96)

The projected mean population immunity following application of three separate campaign calendars is compared to the estimated population immunity in June 2015. 95 % credible interval in parentheses

^a^ Population immunity as of June 20th, 2015

^b^ Population immunity as of April 10th, 2016

b, bivalent; OPV, Oral polio vaccine; t, trivalent

Under the tOPV calendar, all states would have greater than 75 % immunity to type 2 PV before the removal of OPV2 from use in immunization except for Borno, since 16 of 21 districts in the province would remain inaccessible through 2016 due to insecurity under our model.

## Discussion

The Nigeria polio program underwent a restructuring process in October 2012 that led to the creation of the Polio Emergency Operation Center, an assembly of polio public health experts from international development agencies, such as WHO, UNICEF, Red Cross, and CDC, working closely together under the leadership of the Federal Government of Nigeria.

The recent success against polio has been attributed to the widespread use of data for action, innovative methods such as GIS/GPS tracking [18], use of dashboards, and statistical modeling to project population immunity. These population immunity projections and maps provided valuable guidance to the program in determining areas of low population immunity where the PV was likely to take seed and cause an outbreak. By studying these immunity maps, the program was able to take pre-emptive actions to ensure that the best vaccination teams and supervisors were deployed to these weak or vulnerable areas, to ensure that the immunization campaigns were of the highest quality, improve population immunity and, therefore, stave off any PV outbreaks.

In 2012–2013, when PVs were being frequently isolated despite improving campaign quality, population immunity maps 1 were used as advocacy tools to encourage political office holders not to relent on their political and financial support to the program despite setbacks.

Because population immunity maps closely matched the other indices used to assess the quality of supplemental immunization activities, they provided real proof to the GPEI that Nigeria was making progress towards stopping polio transmission despite skepticism on the part of many about the quality of data generated from immunization activities.

Furthermore, over the course of several months, population immunity maps were used as an additional layer of evidence to determine the performance of LGA teams in the implementation of the accountability framework. Thus, LGA programs were more likely to be acknowledged and rewarded if population immunity projections also aligned with other criteria used for performance management.

Our calculations of campaign effectiveness showed steadily increasing quality from 2004–2014, in line with increased focus and resources devoted to the country’s polio program [19, 20]. In Kano, the large improvement in 2014 was correlated with the widespread use of GPS vaccinator tracking [18] and GIS-based micro planning [19] to ensure all settlements are visited by vaccinators. In addition, mobile health camps providing additional routine immunizations, oral rehydration salts, and other medications delivered during vaccination campaigns began to be used at scale in 2014 – especially in Kano.

In contrast, campaign effectiveness showed mixed improvement in northeast states, particularly Borno, comparing 2010 and 2014 results. The northeast has been the center of civil conflict between the extremist group Boko Haram and civil authorities, and accessibility of areas to polio vaccinators has been affected [21]. Our results suggest that efforts to improve campaign effectiveness in the northeast have been impeded, most likely due to regional insecurity.

When comparing our campaign effectiveness estimates with LQAS data, we found a good correlation but a systematic downward bias of our estimates [5], suggesting that the number of doses reported by AFP cases could be systematically under-reported, that the populations captured by AFP are relative underserved compared to those captured by the LQAS surveys, or that the LQAS estimates are biased upwards, e.g. when difficult to access populations are not surveyed.

Similarly, immunity reconstructions show overall improvement in northern Nigeria, particularly for type 1. This is mirrored in case incidence: the last case of type 1 WPV was in July 2014 [22]. However, the explicit link between vaccine usage and our immunity model shows that type 2 results are far more mixed: long periods of no or limited tOPV use result in declining immunity. Efforts to rapidly increase type 2 immunity before cessation of type 2 OPV in April 2016 with increased campaigns are reflected in our immunity results, which also illustrate what may be expected by applying different campaign calendars. These results emphasize lingering concerns over type 2 immunity in Borno.

Our estimates of campaign effectiveness and population immunity rely on the accuracy of AFP data. The varying causes and detection rates of AFP make bias in surveillance data both likely and difficult to quantify [23]. It is possible that AFP data is more effective than traditional cluster surveys in measuring under-served populations, as those people who become paralyzed may seek treatment even if they are not represented within a sampling frame or present at the time of the survey. Conversely, remote populations may not have access to health services and thus not report their cases of paralysis.

Assessing campaign quality from surveillance data is also complicated by the difficulty to recall vaccination dose history, for example in areas which receive many campaigns, or when the child is older. Poor recall in older age groups may be captured by the age-structure model to some extent. In our model, we saw that the estimated campaign effectiveness for older children was lower than for younger children, which could be evidence of inaccurate dose recall.

Our method also relies on the quality and granularity of historical campaign data. In certain special cases, the official campaign calendar may not reflect circumstances when campaigns were cancelled due to accessibility issues (e.g. in Borno state), or when special interventions are conducted to deliver additional doses (e.g. health camps and permanent vaccination teams). In these rare cases, it is possible for our estimates of campaign effectiveness to be biased downward (when campaigns are cancelled) or upward (when vaccination is conducted outside of campaigns).

Explicitly incorporating information about routine immunization (RI) coverage into our methods would improve the accuracy of our estimates of immunity and campaign coverage. Currently, we estimate the vaccine mix received by an AFP case using only the campaign history. This is a good approximation in areas which use tOPV exclusively or where RI coverage is low. In areas where multiple types of vaccines are used (tOPV, mOPV1, mOPV3, bOPV), the accuracy of immunity estimates would be improved by considering RI: increasing the type 2 and decreasing the types 1 and 3. Our estimates of campaign effectiveness assume all received OPV doses are due to vaccination campaigns, such that the ratio of received doses to the number of campaigns exposed to is a measure of campaign effectiveness. In areas where RI coverage is high, multiple OPV doses can be attributed to RI instead of campaigns; incorporating this effect, our estimates of campaign effectiveness would be reduced, and the accuracy of our estimates would be improved. In northern Nigeria, RI coverage is low and the number of vaccination campaigns is high, such that the method presented produces good results.

Going forward, we could also incorporate regional differences and the uncertainty in per-dose vaccine efficacy, and we could attempt to explicitly model within-district heterogeneity in campaign coverage to better capture the effect of chronically missed children.

## Conclusions

Surveillance data is central to GPEI eradication efforts and dosing data in AFP has proven programmatic usefulness [7, 8]. The campaign effectiveness and immunity model presented here advance the use of these data and can help polio eradication efforts.

The use of Bayesian hierarchical modeling allows estimation of campaign quality and immunity at small spatial scales, e.g. LGA. These can be used to highlight effectiveness or immunity gaps in such areas, allowing the possibility of sharper programmatic focus.

Previous methods of polio immunity modeling have relied on time binning, with hierarchical modeling of binned observations to handle low sampling rates for small area estimation [9]. This method of immunity reconstruction and forecasting improves on past methods by explicitly linking campaign effectiveness and campaign event history, the latter including the vaccine used. The result is immunity estimation that responds dynamically to campaign events – past and proposed – to yield immunity by serotype. This is useful in the polio eradication endgame for elimination modeling for multiple polio types, evaluation of prospective campaign calendars, and changes in vaccine usage. The methods may also be partially extensible to other vaccine preventable diseases that rely on campaigns to build population immunity.

## Additional file

Additional file 1: Supplement: Analysis of vaccination campaign effectiveness and population immunity to support and sustain polio elimination in Nigeria. (DOCX 478 kb)

## Abbreviations

AFP: Acute flaccid paralysis
bOPV: Bivalent oral polio vaccine
cVDPV: Circulating vaccine-derived poliovirus
GPEI: Global Polio Eradication Initiative
LGA: Local government area
LQAS: Lot quality assurance sampling
mOPV: Monovalent oral polio vaccine
NP-AFP: Non-polio acute flaccid paralysis
OPV: Oral polio vaccine
PV: Poliovirus
RI: Routine immunization
SIA: Supplemental immunization activity
tOPV: Trivalent oral polio vaccine
WPV: Wild poliovirus

## Footnotes

1 Using older methods, for example [9].
