# Supplementary material for: Analysis of vaccination campaign effectiveness and population immunity to support and sustain polio elimination in Nigeria
Source: BMC Med. 2016 Mar 30;14:60. doi: 10.1186/s12916-016-0600-z (PMC4812602; doi:10.1186/s12916-016-0600-z)
Supplement: Additional file 1: — Supplement: Analysis of vaccination campaign effectiveness and population immunity to support and sustain polio elimination in Nigeria. (DOCX 478 kb) [file 12916_2016_600_MOESM1_ESM.docx]

Supplement: Analysis of vaccination campaign effectiveness and population immunity to support and sustain polio elimination in Nigeria

Alexander M Upfill-Brown, Arend Voorman, Guillaume Chabot-Couture, Faisal Shuaib, Hil M Lyons

# Methods

## Model for campaign effectiveness

Campaign effectiveness is the coverage as estimated by NPAFP data and the campaign history. We have modeled coverage, a value constrained between 0 and 1, on the convenient logit scale.

We specify a flexible hierarchical model of campaign effectiveness to account for temporal patterns and between-LGA differences. The model is fit separately for each state, and for notational convenience we describe the model as it applies to a single state. Let $p_{jta}$be the campaign effectiveness for LGA $j$, year $t$, and age stratum $a$. Campaign effectiveness is assumed to be constant for each year of the campaign. We model $p_{jta}$ with

$$\mathrm{logit}\left( p_{jta} \right)=\beta_{a}+b_{j}+u_{t}+v_{jt},$$

where $\beta_{a}$ is a fixed effect by age stratum, $b_{j}\sim N\left( 0,\sigma^{2} \right)$ is a random effect for LGA and $\left[ u_{1},\ldots,u_{T} \right]^{T}\sim N_{T}\left( \boldsymbol{0},\boldsymbol{\Sigma}\left( \sigma_{u}^{2} \right) \right)$ and $\left[ v_{j1},\ldots,v_{jT} \right]^{T}\sim N_{T}\left( \boldsymbol{0},\boldsymbol{\Sigma}\left( \sigma_{v}^{2} \right) \right)$ are first order normal random walks for LGA and state.

## Error modeling and heterogeneity

When doses are observed without error, conditional on a sequence of campaign coverage $p_{1},\ldots,p_{k}$with independent participation, the distribution of doses follows a Poisson-Binomial distribution[1]. The mean and variance of the number of doses $y$ are

$$E\left( y | p_{1},\ldots,p_{k} \right)=\sum_{i=1}^{k} p_{i}$$

$$Var\left( y | p_{1},\ldots,p_{k} \right)=\sum_{i=1}^{k} p_{i}\left( 1-p_{i} \right),$$

both arising from the conditional independence of a sum of Bernoulli trials.

To allow for a more flexible mean-variance relationship, we use a Negative Binomial distribution to model error in reported doses per child, where the expected value (mean) is the sum of campaign effectiveness across campaigns experienced by the child[2]. The mass function may be written

$$P\left( y | p_{1},\ldots,p_{k} \right)=\left( \begin{matrix} y+\theta-1 \\ y \end{matrix} \right)\left( \frac{\mu}{\mu+\theta} \right)^{y}\left( \frac{\theta}{\mu+\theta} \right)^{\theta},$$

with $\mu=\sum_{i} p_{i}$ and $\theta>0$. The parameter $\theta$ controls the overall level of dispersion, with

$$Var\left( y | \mu,\theta\right)=\mu+\frac{\mu^{2}}{\theta}.$$

The Negative Binomial formulation has uniformly larger variance than the Poisson-Binomial formulation, as

$$\sum_{i=1}^{k} p_{i}\left( 1-p_{i} \right)<\sum_{i=1}^{k} p_{i}=\mu< \mu+\frac{\mu^{2}}{\theta}.$$

While relative variance to the Poisson Binomial is not preserved, the Negative Binomial distribution provides flexibility in the amount of overdispersion while preserving the mean.

Note that use of the negative binomial attributes all the excess variation over that induced by the hierarchical mean model to error in dose reporting. Other forms of heterogeneity, for example, differential coverage for the chronically missed children are possible, but likely come with identifiability issues. That is, it may be difficult to disaggregate dose recall error from true heterogeneity in absence of a known model for dose recall. We do not attempt to address that in this analysis.

## Random walk models

The discrete first order random walk is a Gaussian Markov random field (GMRF) characterized in this analysis by independent and identically distributed increments between time intervals[3]. That is, if $u_{t+1}-u_{t}\sim N\left( 0, \sigma^{2} \right)$, then $u_{t+1}|u_{t}\sim N\left( u_{t},\sigma_{u}^{2} \right)$. This implies a certain smoothness or coherence in coverage from year to year.

The joint density of the $T$ random walk terms may be written in terms of the differences

$$f\left( \boldsymbol{u} \right)=\left( 2\pi\sigma_{u}^{2} \right)^{-\frac{T-1}{2}}\exp\left\{ -\frac{1}{2\sigma_{u}^{2}}\left( \sum_{t=1}^{T-1} \left( u_{t+1}-u_{t} \right)^{2} \right) \right\}\boldsymbol{1}_{\left[ \sum_{t=1}^{T} u_{j}=0 \right]}\boldsymbol{.}$$

This is a proper density due to the constraint $\sum_{j=1}^{n} u_{j}=0$. An alternative representation is with precision $\boldsymbol{Q}=\frac{1}{\sigma_{u}^{2}}\boldsymbol{R}$ where $\boldsymbol{R}$ is called the structure matrix.

Relevant to MCMC sampling of the posterior distribution is specification of a sampling procedure for this density that satisfies the constraint $\sum_{j=1}^{n} u_{j}=0$. One form utilizes the Cholesky factorization for a rank $n-1$ positive definite submatrix of the generalized inverse of $\boldsymbol{Q}$.

That is, let $\boldsymbol{\Sigma}$ be the generalized or Moore-Penrose inverse of $\boldsymbol{Q}$, the covariance matrix of $\boldsymbol{u}=\left[ u_{1},\ldots u_{T} \right]^{T}$. Consider any $\left( T-1 \right)$-dimensional subset of $\boldsymbol{\Sigma}$, denoted here $\boldsymbol{\Sigma}_{T-1}$, which will be positive definite in the random walk case. The Cholesky factorization is a (lower) triangular matrix $L$ such that $\boldsymbol{L}\boldsymbol{L}^{T}=\boldsymbol{\Sigma}_{T-1}$. Then one sampling strategy consists of the following:

Step 1: Sample $z_{t}\sim N\left( 0,1 \right)$, $t=1,\ldots,T-1$.

Step 2: Set $\boldsymbol{u}_{\left( \boldsymbol{-i} \right)}=\boldsymbol{Lz}$ where $i$ is the index of the excluded element of $\boldsymbol{u}$determining the sub-matrix $\boldsymbol{\Sigma}_{n-1}$ ($i$-th row and columns removed). $\boldsymbol{u}_{\left( \boldsymbol{-i} \right)}\sim MVN\left( 0,\boldsymbol{\Sigma}_{T-1} \right)$.

Step 3: Set $u_{i}=-\sum_{\left( j\neq i \right)} u_{j}$ to satisfy the constraint $\sum_{j=1}^{n} u_{j}=0$.

In practice, sampling in STAN is a lower level function guided by the specification of (log) densities. STAN includes a convenient specification for the multivariate normal distribution using the Cholesky factorization. In the case of the random walk, and unscaled Cholesky factorization may be computed in advance based on inverting the structure matrix $R$ and rescaling with the appropriate variance. Note that in our setting, there are state and LGA level random walks, but as they cover the same time period the structure matrix $R$ is identical as is the corresponding Cholesky factorization. For an alternate sample strategy, see [3].

## Immunity estimation

Here, we describe the procedure for estimating immunity from campaign coverage. Without loss of generality, we limit the discussion to a single polio serotype. Suppose a child in LGA $j$ experiences their first campaign in year $t$ and age $a$ with no routine immunization. To become immune, the child must be covered in the campaign and seroconvert. The probability of neither of these events occurring is $1-p_{jta}\phi_{jt}$ where $\phi_{jt}$ the probability of take (efficacy) for the particular vaccine used.

To generalize, suppose at time $\tilde{t}$, a child of age $\tilde{a}$ has experienced campaigns in years $t_{1}\leq\ldots\leq t_{k}$ and corresponding age stratums $a_{1}\leq\ldots\leq a_{k}.$ With independent participation in campaigns and independent seroconversion, the probability $I(t,a)$ of vaccine-based seroconversion for the child is

$$I_{j}\left( \tilde{t},\tilde{a} \right)=1-\prod_{m=1\ldots k} \left( 1-p_{jt_{m}a_{m}}\phi_{jt_{m}} \right).$$

Note that immunity $I_{j}(\tilde{t},\tilde{a})$ is a function of the continuous variables $\tilde{t}$ and $\tilde{a}$, whereas campaign effectiveness $p_{jta}$ is indexed by year $t$ and age stratum $a.$ In the model employed, children of an identical age living in the same LGA have the same probability of being immune due to identical campaign exposure. Therefore, population immunity over an age group distribution $F$ may be obtained by taking the expectation (i.e. integrating) $I_{j}(\tilde{t},\tilde{a})$ with respect to the distribution $F$. That is

$$I_{j}\left( \tilde{t} \right)=\int I_{j}\left( \tilde{t},\tilde{a} \right)dF\left( \tilde{a} \right).$$

We compute the time course of immunity through an efficient dynamic programming algorithm. We start with $I\left( \tilde{t},0 \right)=0$, i.e. newborn children are not immune. Now, suppose $I_{j}(\tilde{t},\tilde{a})$ is known for fixed $\tilde{t}$ and all ages $\tilde{a}$. Then, for sufficiently small $\delta$ we can compute immunity at future time points using the equations:

$$I_{j}\left( \tilde{t}+\delta, \tilde{a}+\delta\right)=I_{j}\left( \tilde{t},\tilde{a} \right) if no campaigns occured in the interval (\tilde{t},\tilde{t}+\delta];$$

$$I_{j}\left( \tilde{t}+ \delta, \tilde{a}+\delta\right)=1-\left( 1-I_{j}\left( \tilde{t},\tilde{a} \right) \right)\left( 1-p^{*}\phi^{*} \right) if a campaign occured in the interval (\tilde{t},\tilde{t}+\delta],$$

where $p^{*}$ and $\phi^{*}$ are the appropriate campaign coverage and vaccine efficacy for the campaign occurring in the interval $(\tilde{t},\tilde{t}+\delta]$.

In practice, we keep track of immunity in a discrete set of ages from 0 to 5 years old, separated by $\delta$ = 7 days. Then, starting in January 1, 2000 with all age groups being un-immune, we can calculate immunity in 7 day increments, so that the updates $I_{j}(\tilde{t}+\delta, \tilde{a}+\delta)$ yield immunity in the next oldest age group. We then use a discrete approximate to $I_{j}\left( \tilde{t} \right)=\int I_{j}\left( \tilde{t},\tilde{a} \right)dF\left( \tilde{a} \right)$, where $F$is the uniform distribution on 6-59 month olds.

## Uncertainty propagation for immunity

The output of an MCMC procedure is a set of samples that approximate the posterior distribution of the parameters. The particular parameters of interest are campaign effectiveness parameters by age, which are composed of various random and fixed effects.

In addition, population immunity $I_{j}(\cdot)$ is a function of campaign effectiveness, and so its posterior distribution can be approximated by applying the immunity calculations to posterior samples. Let $I_{j}^{(k)}= I_{j}^{\left( k \right)}\left( \tilde{t} \right), i=1,\ldots,K$ be the population immunity estimates for a particular time point $\tilde{t}$, based on the $K$ MCMC samples of the parameters. Then the posterior expectation and variance for population immunity are given by

$$\tilde{E(I_{j}|\boldsymbol{Y})}\boldsymbol{=}\frac{1}{K}\sum_{k=1}^{K} I_{j}^{\left( k \right)} \mathrm{and}$$

$$\tilde{V\left( I_{j} | \boldsymbol{Y} \right)}\boldsymbol{=}\frac{1}{K-1}\sum_{k=1}^{K} \left( I_{j}^{\left( k \right)}-\tilde{E\left( I_{j} | \boldsymbol{Y} \right)} \right)^{2}.$$

Similarly, posterior quantiles for population immunity may be available as well.

## Bayesian hierarchical model specification

Likelihood, prior, and hyperprior specification is as follows. Let $k$ refer to the $k$-th child, and $y_{jk}$ the doses experienced by the $k$-th child in LGA $j$. Then

$$y_{jk}|\mu_{jk},\theta\sim NegBinomial\left( \mu_{jk},\theta\right)$$

$$\mu_{jk}=\sum p_{jt,a\left( k,t \right)},$$

where the sum is taken over campaigns experienced by child, where effectiveness may depend on age of the child at the time of the campaign. This is slightly informal notation as there is no index for campaigns provided that would serve to indicate which campaigns were experienced by a child.

Then,

$$\mathrm{logit}\left( p_{jta} \right)=\alpha+\beta_{a}+b_{j}+u_{t}+v_{jt}$$

$$\alpha\sim N\left( 0,2 \right)$$

$$\beta_{a}\sim N\left( 0,1 \right)$$

$$b_{j}|\sigma^{2}\sim N\left( 0,\sigma^{2} \right)$$

$$\boldsymbol{u}|\sigma_{u}^{2}\sim MVN\left( 0,\boldsymbol{\Sigma}\left( \sigma_{u}^{2} \right) \right)$$

$$\boldsymbol{v}_{\boldsymbol{j}}|\sigma_{v}^{2}\sim MVN(0,\boldsymbol{\Sigma}\left( \sigma_{u}^{2} \right))$$

$$\pi\left( \theta\right)\propto1$$

$$\sigma^{2}\sim InvGamma\left( 0.1,0.1 \right)$$

$$\sigma_{u}^{2}\sim InvGamma\left( 0.1,0.1 \right)$$

$$\sigma_{v}^{2}\sim InvGamma\left( 0.1,0.1 \right)$$

Note that there are 5 age categories by age of life; the base category, <1 year olds is represented by $\alpha$, with $\beta_{a}$ representing age effects of the other 4 age groups relative to the base category. The priors for these parameters combine to center effectiveness at 50% (median). The choice of expression $\pi\left( \theta\right)\propto1$ indicates a improper flat prior for the overdispersion parameter of the Negative Binomial.

## Vaccine Per-Dose Efficacy

Per dose efficacies for OPV were taken from [5] and can be found in Table S1. These values were estimated using NP-AFP data in northern Nigeria, and are therefore consistent with the data used in this study.

**Table S1 – Polio vaccine efficacies used to estimate population immunity.**

|  | tOPV | bOPV | mOPV1 | mOPV3 | IPV |
| --- | --- | --- | --- | --- | --- |
| Type 1 | 19.2 | 29.9 | 28.8 | - | 80.0 |
| Type 2 | 48.9 | - | - | - | 90.0 |
| Type 3 | 17.7 | 24.0 | - | 40.9 | 80.0 |

IPV has been used very recently in Nigeria in vaccination campaigns in highest risk areas. The inclusion of IPV in our standard calculation is complicated by the fact that IPV efficacy against paralysis is varies depending on previous OPV exposure. Seronegative children who have received OPV previously seroconvert at a higher rate than OPV-naive children receiving IPV as a first dose [6-10]. If children are seronegative (i.e. no detectable antibody) but have a history of OPV exposure, they seroconvert at a much higher rate.

Because OPV is given to children at the same time that they receive IPV in vaccination campaigns, and because children have been exposed to OPV regularly in these areas, we used an efficacy for IPV derived from three studies on IPV seroconversion following IPV exposure [8-10]. Average per dose efficacies from these studies were 82.3 (126/153), 92.3 (169/181), and 85.2 (305/358) for types 1, 2, and 3, respectively. There is uncertainty surrounding these estimates, so we treat the values used in this study as rough guides.

# Results

## Kano Model Selection

In Kano, we tried four different versions of the campaign effectiveness model: 1) a model with no age effect and fixed between-district variation over time, 2) a model allowing for district-level variation over time, 3) a model similar to (1) with only an age effect added, and 4) a model with both the district-level random walk and age effects. The coverage model that allows for the most complexity – district-level variation over time, age structure – provides the best fit in terms of DIC (Table S2).

In both models with the age effects (models 3 and 4), campaign effectiveness was estimated to be lowest in the 24-35 month age group (2 year olds) relative to 6-11 month olds. 12-23 month olds experienced the lowest reduction relative to 6-11 month olds in campaign effectiveness.

**Table S2 – Kano campaign effectiveness model results**, posterior 95% credible intervals in parentheses

|  |  | (1) | (2) | (3) | (4) |
| --- | --- | --- | --- | --- | --- |
|  | Variable | Base | District Random Walk | Age-based Participation | Full |
| **Fixed Effects** | Intercept | -1.52 | -1.54 | -1.09 | -1.11 |
|  |  | (-1.64, -1.38) | (-1.67, -1.41) | (-1.24, -0.93) | (-1.27, -0.94) |
| *Age-based Participation* | 12-23 M | - | - | -0.22 | -0.2 |
|  |  | - | - | (-0.51, 0.06) | (-0.48, 0.07) |
|  | 24-35 M | - | - | -1.8 | -1.82 |
|  |  | - | - | (-2.61, -1.27) | (-2.61, -1.3) |
|  | 36-47 M | - | - | -0.86 | -0.82 |
|  |  | - | - | (-1.26, -0.51) | (-1.23, -0.48) |
|  | 48-59 M | - | - | -1.13 | -1.04 |
|  |  | - | - | (-2.01, -0.45) | (-1.95, -0.41) |
| **Random Effects** | $\sigma^{2}$ | 0.23 | 0.19 | 0.21 | 0.19 |
|  |  | (0.17, 0.3) | (0.02, 0.28) | (0.15, 0.29) | (0.13, 0.27) |
|  | $\sigma_{u}^{2}$ | 0.3 | 0.32 | 0.29 | 0.3 |
|  |  | (0.07, 0.88) | (0.08, 0.99) | (0.08, 0.82) | (0.08, 0.88) |
|  | $\sigma_{v}^{2}$ | - | 0.08 | - | 0.06 |
|  |  | - | (0.04, 0.13) | - | (0.03, 0.11) |
| **Summary Statistics** | $\theta$ | 4.59 | 5.56 | 5.22 | 6.43 |
|  |  | (4.02, 5.15) | (4.92, 6.23) | (4.65, 5.85) | (5.65, 7.29) |
|  | DIC | 13459.5 | 13373.9 | 13264.9 | 13184.8 |
|  | N | 3159 | 3159 | 3159 | 3159 |
|  | | | |  |  |

## Northern state campaign effectiveness

Across all states we found that 12-23 month olds had the smallest reduction in campaign effectiveness relative to the youngest cohort (Figure S1). Reduction in campaign effectiveness plateaued in the older age groups with reduction relative to 6-11 month olds being relatively similar. In all states, model fit as measured by DIC was lowest in the full model compared to the other models. For this reason, we used the full model to estimate campaign effectiveness and reconstruct population immunity across northern states.


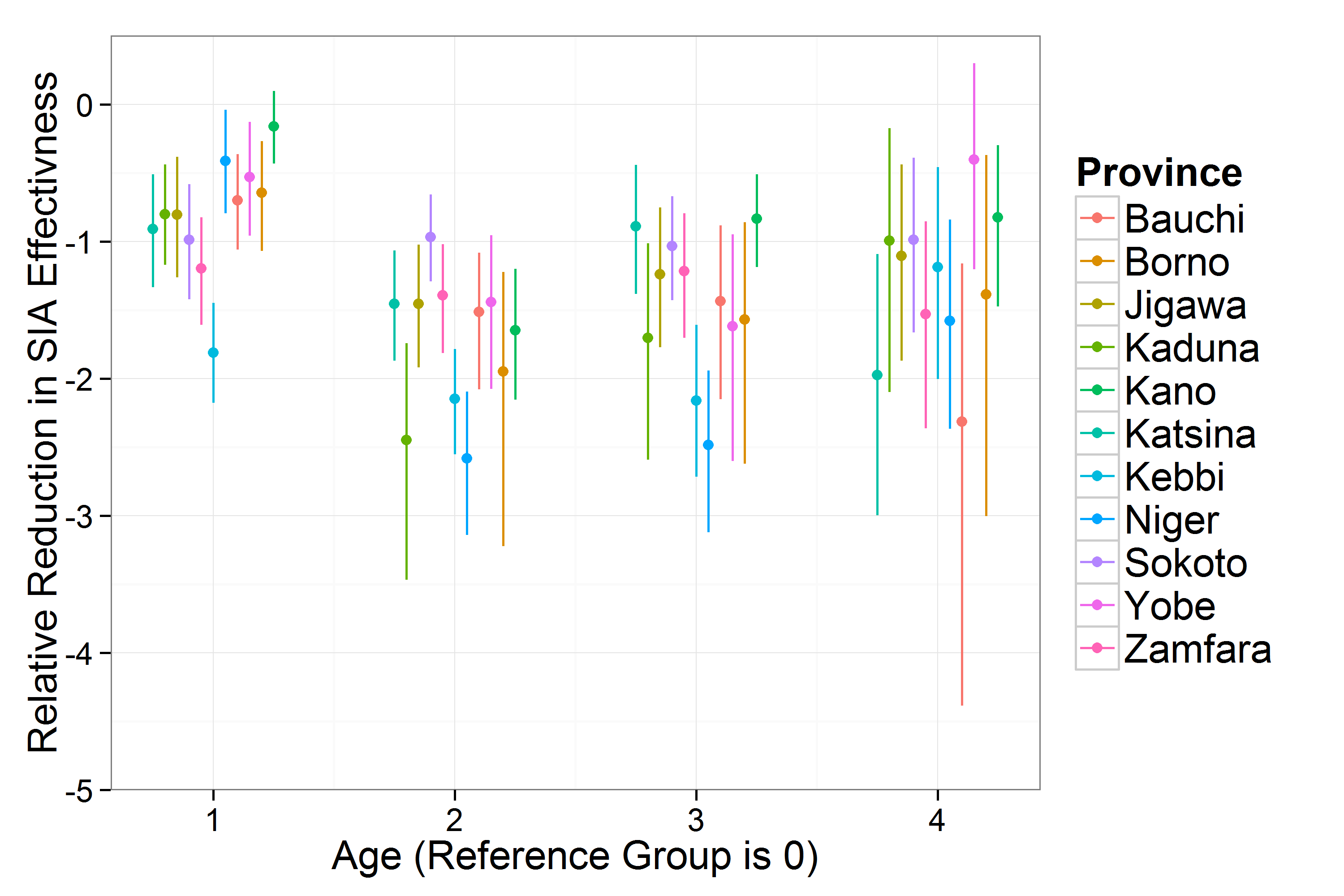


Figure S1 - Age effect estimates across all states from the full model, model 4 in Table S1. Estimates are on the log odds scale; bars represent posterior 95% credible intervals. Reference group is the 6-11 month age cohort. Age group 1 corresponds to 12-23 month-olds, age group 2 to 24-35 month-olds, age group 3 to 36-47 month olds and age group 4 to 48-59 month olds.

Using district population estimates from the 2006 Nigerian census, we combined district campaign effectiveness estimates into province-level averages (Table S3). The changes between effectiveness in 2014 compared to 2010 were very large in Jigawa, Kano, Katsina, Sokoto and Zamfara (mean improvement > 40%). Bauchi and Borno had the smallest estimated improvements in campaign effectiveness over this period (mean improvement < 10%). Campaign effectiveness remained below 50% in Bauchi, Borno, Kaduna and Yobe.

Table S3 - Estimates of northern state campaign effectiveness.

| **Province** | **2010** | | **2014** | |
| --- | --- | --- | --- | --- |
|  | Mean | CI | Mean | CI |
| Bauchi | 0.27 | (0.23 - 0.31) | 0.34 | (0.28 - 0.42) |
| Borno | 0.19 | (0.16 - 0.22) | 0.26 | (0.20 - 0.33) |
| Jigawa | 0.20 | (0.16 - 0.25) | 0.84 | (0.70 - 0.97) |
| Kaduna | 0.29 | (0.25 - 0.34) | 0.41 | (0.32 - 0.50) |
| Kano | 0.20 | (0.17 - 0.23) | 0.75 | (0.64 - 0.86) |
| Katsina | 0.23 | (0.18 - 0.28) | 0.81 | (0.70 - 0.91) |
| Kebbi | 0.32 | (0.26 - 0.40) | 0.59 | (0.49 - 0.69) |
| Niger | 0.42 | (0.36 - 0.47) | 0.59 | (0.47 - 0.68) |
| Sokoto | 0.21 | (0.17 - 0.25) | 0.70 | (0.62 - 0.79) |
| Yobe | 0.23 | (0.18 - 0.28) | 0.43 | (0.33 - 0.54) |
| Zamfara | 0.29 | (0.24 - 0.36) | 0.71 | (0.59 - 0.83) |

## LQAS comparison

Following a vaccination campaign, independent surveyors go to 6 randomly chosen villages within a district and check the finger-marking of 10 children[4]. Only a subset of districts participating in a vaccination campaign are visited by LQAS surveyors. In the course of a year, most districts are visited by surveyors at least one time. We include LQAS surveys from 2009 through 2015 in our analysis.

5770 LQAS surveys were matched (by SIA date and by LGA) with calculated coverage, spanning years from 2009 to 2015, and in the following states of Nigeria: Bauchi, Borno, Jigawa, Kaduna, Kano, Katsina, Kebbi, Niger, Sokoto, Yobe, and Zamfara. Since calculated campaign effectiveness is constant in a given year, all measurements for a given LGA within a year will be matched to a single calculated coverage.

**Table S4 - Average bias and correlation coefficient, by state, of matched LQAS surveys and calculate campaign effectiveness**

| **State** | **Average bias** | **Correlation coefficient** | **# of lots matched** |
| --- | --- | --- | --- |
| Bauchi | -52% | 0.29 | 368 |
| Borno | -54% | 0.28 | 433 |
| Jigawa | -23% | 0.41 | 562 |
| Kaduna | -44% | 0.29 | 458 |
| Kano | -31% | 0.64 | 977 |
| Katsina | -21% | 0.39 | 762 |
| Kebbi | -34% | 0.32 | 438 |
| Niger | -29% | 0.24 | 452 |
| Sokoto | -23% | 0.46 | 549 |
| Yobe | -44% | 0.48 | 407 |
| Zamfara | -30% | 0.33 | 364 |

The bias between calculated coverage and LQAS coverage varies from state to state, from -21% in Katsina to -54% in Borno (Table S4). The correlation coefficient also varies, from 0.24 in Niger state to 0.64 in Kano state. All p-values are less than 0.001.

**Table S5 -Average bias and correlation coefficient, by age group, of matched LQAS surveys and calculate campaign effectiveness**

| **Age group** | **Average bias** | **Correlation coefficient** | **P-val** |
| --- | --- | --- | --- |
| 0-1 year olds | -15% | 0.45 | <0.001 |
| 1-2 | -28% | 0.43 | <0.001 |
| 2-3 | -43% | 0.38 | <0.001 |
| 3-4 | -38% | 0.38 | <0.001 |
| 4-5 | -37% | 0.39 | <0.001 |

When the coverage per age group is compared with the matched LQAS coverage (Table S5), it is found that the coverage of the 0-1 year old age group has the smallest bias and the greatest correlation coefficient. This could be due to better dose recall in this age group.


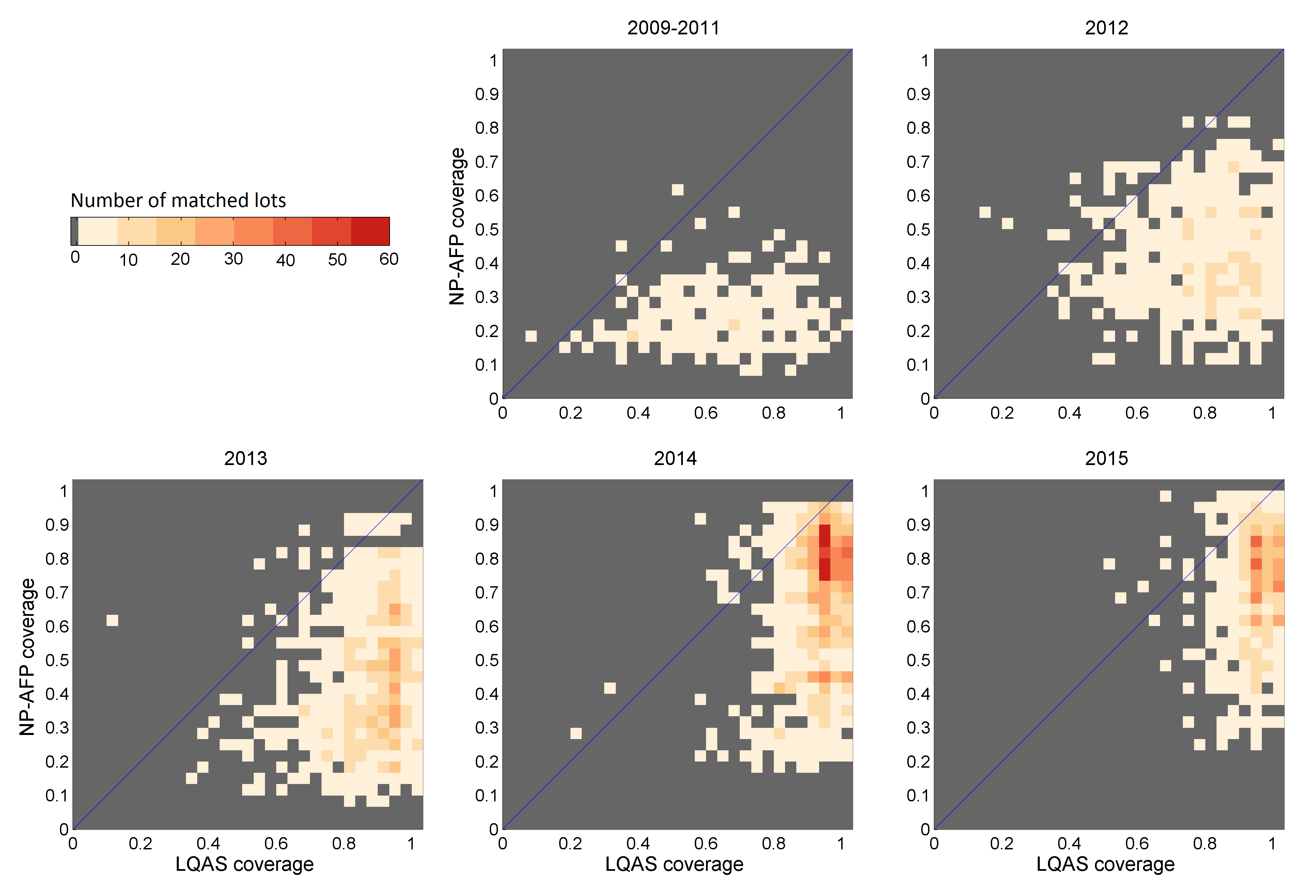


Figure S2 - Two-dimensional histogram of LQAS survey results and the matched NP-AFP calculated campaign effectiveness. Each colored square represents a pair of values for LQAS coverage and NP-AFP coverage. The color of each square, from light red to dark red, is the number of matched lots; gray is zero matched lots. If matched lots are distributed along the diagonal, there is good agreement between LQAS and NP-AFP measures of coverage. Matched lots away from the diagonal indicate a bias between the two measures of coverage. The number of lots matched is different in different time periods.

In Figure S2, we show the distribution of matched lots, in the two dimensional space of campaign effectiveness and LQAS coverage, for different time periods. We see that there is significant scatter in the early years, which is reduced in 2014 and 2015.


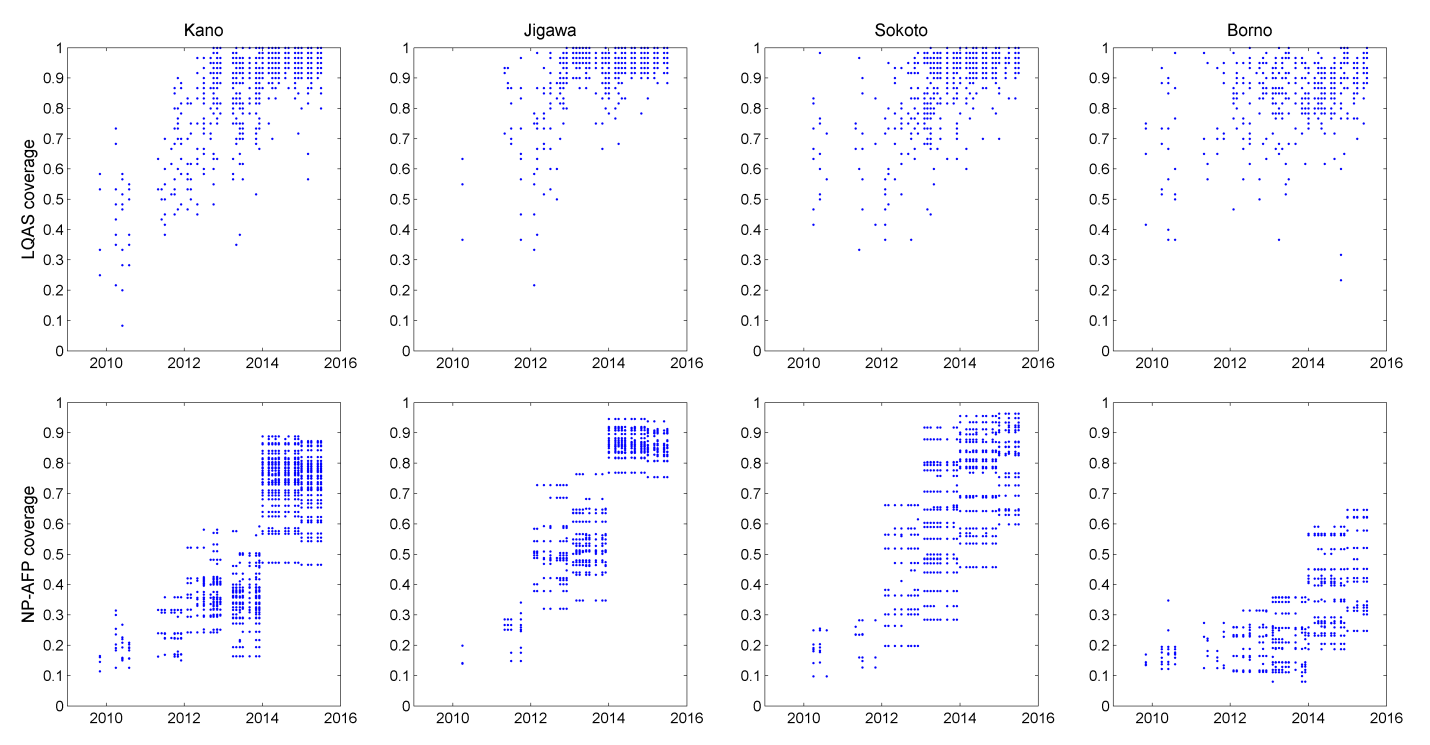


Figure S3 - Examples of the comparison between NP-AFP coverage (estimated campaign effectiveness) and LQAS survey results for selected states in Northern Nigeria. Each blue dot in the top-row figures represent one LQAS measurement. These LQAS lots are matched to calculated NP-AFP coverage estimates in the bottom-row figure. Some blue dots overlap other blue dots.

In figure S3, we show the time trends of the campaign effectiveness (NP-AFP coverage) and LQAS coverage for four states: Kano, Jigawa, Sokoto, and Borno (Figures S2 and S3). Good agreement is found between the trends in LQAS coverage and in NP-AFP coverage (estimated campaign effectiveness). NP-AFP coverage is generally less than the LQAS coverage estimates; Borno is an example where this difference can be clearly seen.

## Relationship between reconstructed immunity and confirmed cases

Reconstructed population immunity is significantly related to the presence and number of polio (WPV1 and cVDPV2) cases across Nigeria. 99.1% of districts in northern Nigeria with greater than 80% type 1 immunity did not report a WPV case in the following six months (Figure S4,A). The average type 1 immunity of districts that reported a case in the following six months was 29%, while the average type 1 immunity in districts that did not report a case was 53% (p<0.001, Welch two sample t-test). For cVDPV2 cases, 99.4% of districts in the north with greater than 50% type 2 immunity did not report a case in the subsequent six-month period (Figure S4,B). Average type 2 immunity was 27% in districts that reported a cVDPV2 case in the following six month, compared to 39% in districts that did not report a case (p<0.001).


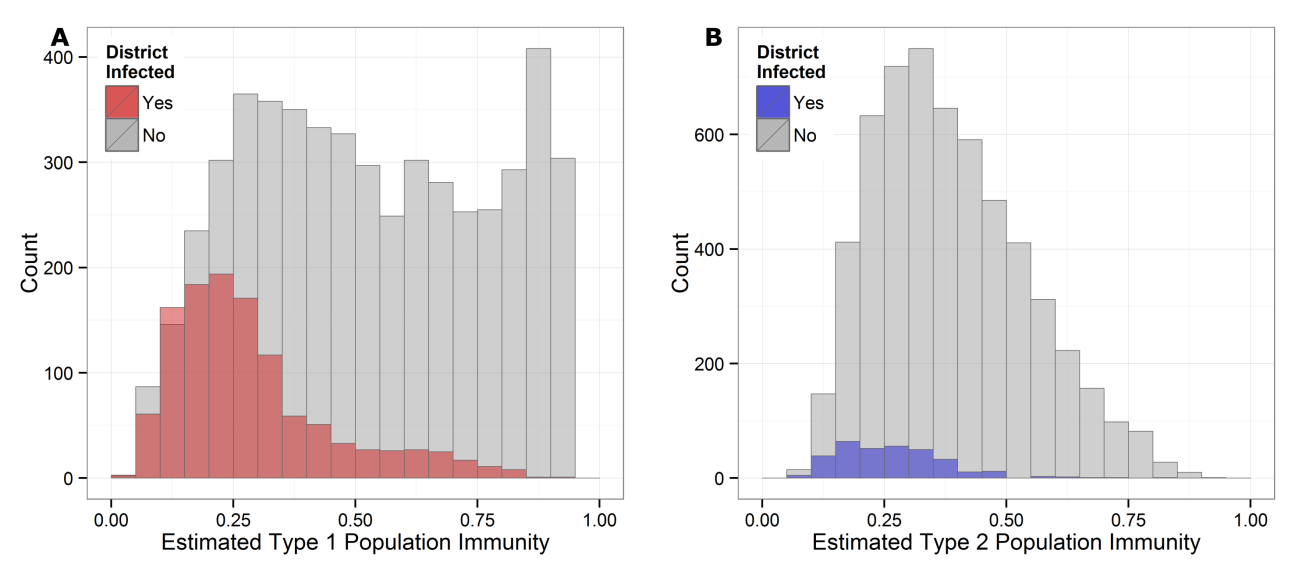


Figure S4 - Histograms of district population immunity relative to occurrence of confirmed cases. Estimated district-level type 1 (A) and type 2 (B) immunity separated by the occurrence of WPV1 or cVDPV2 cases, respectively, in the following six month period. Histograms are overlaid such that red (A) corresponds to the distribution of type 1 immunity in districts reporting a WPV1 case and blue (B) corresponds to the distribution of type 2 immunity in districts reporting a cVDPV2 case. Gray corresponds to the distribution of estimated immunity in districts that did not report any confirmed PV cases.

Another NP-AFP indicator commonly used by the polio program is the fraction of NP-AFP cases in a district that report receiving no doses of OPV. Those districts with a higher zero-dose fraction are considered to have higher vulnerability and therefore greater risk of polio transmission. For the same provinces used in the campaign effectiveness model, we smoothed NP-AFP zero-dose fractions by district in six-month periods using a Spatial Bayesian binomial model with random effects and random walks for both province and district. The average zero dose fraction was 23% in districts that reported at least on WPV1 case in the six month window and 11% in districts that did not report a WPV1 case (p<0.001). For cVDPV2, the average zero-dose fraction in districts reporting a cases was 18%, while it was 13% is districts not reporting any case (p<0.001).

We compared the relative strength of association between estimated population immunity and smoothed zero-dose fraction by comparing the AIC from logistic regression models with each indicator as the sole predictor of the presence or absence of confirmed cases in a six month window. For WPV1 cases, the AIC for the model with type 1 population immunity was 4991.1, while the model with zero-dose fraction had an AIC of 5410.7. For cVDVP2 cases, the AIC for the model with type 2 population immunity was 2248.3, while the model with zero-dose fraction had an AIC of 2457.9. In both instances, estimated population immunity was a better predictor of the presence of confirmed cases.

# References

1. Hong Y: **On computing the distribution function for the Poisson binomial distribution**. *Comput Stat Data Anal* 2013, **59**:41–51.

2. Stan Development Team: **Stan: A C++ Library for Probability and Sampling, Version 2.5.0**. 2014.

3. Rue H, Held L: *Gaussian Markov Random Fields: Theory and Applications*. CRC Press; 2005.

4. Organization WH: **Assessing vaccination coverage levels using clustered Lot Quality Assurance Sampling: field manual version edited for the Global Polio Eradication Initiative (GPEI)**. *Geneva, Switz* 2012.

5. Mangal TD, Aylward RB, Mwanza M, Gasasira A, Abanida E, Pate MA, Grassly NC: **Key issues in the persistence of poliomyelitis in Nigeria: a case-control study.** *Lancet Glob Health* 2014, **2**(2):e90-7.

6. Resik S, Tejeda A, Sutter RW, Diaz M, Sarmiento L, Alemañi N, Garcia G, Fonseca M, Hung LH, Kahn AL, Burton A, Landaverde JM, Aylward RB: **Priming after a fractional dose of inactivated poliovirus vaccine**. *N Engl J Med* 2013, **368**(5):416-24.

7. Grassly NC: **Immunogenicity and effectiveness of routine immunization with 1 or 2 doses of inactivated poliovirus vaccine: systematic review and meta-analysis.** *J Infect Dis* 2014, **210 Suppl 1**:S439-46.

8. Moriniere BJ, van Loon FP, Rhodes PH, Klein-Zabban ML, Frank-Senat B, Herrington JE, Pallansch MA, Patriarca PA: **Immunogenicity of a supplemental dose of oral versus inactivated poliovirus vaccine.** *Lancet* 1993, **341**(8860):1545-50.

9. Estívariz CF, Jafari H, Sutter RW, John TJ, Jain V, Agarwal A, Verma H, Pallansch MA, Singh AP, Guirguis S, Awale J, Burton A, Bahl S, Chatterjee A, Aylward RB: **Immunogenicity of supplemental doses of poliovirus vaccine for children aged 6-9 months in Moradabad, India: a community-based, randomised controlled trial.** *Lancet Infect Dis* 2012, **12**(2):128-35.

10. Jafari H, Deshpande JM, Sutter RW, Bahl S, Verma H, Ahmad M, Kunwar A, Vishwakarma R, Agarwal A, Jain S, Estivariz C, Sethi R, Molodecky NA, Grassly NC, Pallansch MA, Chatterjee A, Aylward RB: **Efficacy of inactivated poliovirus vaccine in India.** *Science* 2014, **345**(6199):922-5.
